# That rising obesity levels will greatly add to the burden of cancer: misconceptions I

**DOI:** 10.1038/sj.bjc.6606024

**Published:** 2011-01-04

**Authors:** B J Cairns, T Y O Yang, V Beral

**Affiliations:** 1 Cancer Epidemiology Unit, University of Oxford, Richard Doll Building, Roosevelt Drive, Oxford OX3 7LF, UK

Projections of future obesity levels, based on the well-documented increases during the twentieth century (James *et al*, 2001), have been described as a potential ‘public health catastrophe’ (James *et al*, 2004). Cancer is often highlighted as one of the major health problems linked to excess adiposity. However, most of the obesity-related burden of disease comes from diabetes and cardiovascular disease: only about one-tenth is estimated to be due to cancer (Allender and Rayner, 2007).

Obesity is associated with increases in cancer at a number of sites (Calle and Kaaks, 2004; Reeves *et al*, 2007; Renehan *et al*, 2008; Prospective Studies Collaboration, 2009). Overall, a 1 kg m^−2^ increase in body mass index (BMI) is associated with an approximately 1% increase in incidence for all cancers combined (based on data from women; Reeves *et al*, 2007) and about a 2% increase in total cancer mortality (based largely on data from men; Prospective Studies Collaboration, 2009). Endometrial cancer and oesophageal adenocarcinoma are the malignancies most strongly related to obesity, each being associated with about a 10% increase in incidence per 1 kg m^−2^ increase in BMI. There are weaker associations with postmenopausal breast cancer, colon cancer in men, renal cell carcinoma and certain other cancers. In contrast, for premenopausal breast cancer, lung cancer and squamous cell carcinoma of the oesophagus, risk decreases with increasing BMI (Renehan *et al*, 2008).

During the latter half of the twentieth century, the mean BMI of many Western populations increased by around 1 kg m^−2^ per decade (Prospective Studies Collaboration, 2009), although the rate of increase now appears to be slowing (Cairns *et al*, 2009; Joint Health Surveys Unit, 2009; Brown *et al*, 2010; Howel, 2010) (see Figure 1). We might expect, other things being equal, that the 1 kg m^−2^ per decade increase in BMI should have resulted in 1–2% per decade increases in overall cancer incidence and mortality, although site-specific trends will have varied. This increase is small compared with the effects of changes in other risk factors, such as smoking, and improved cancer detection and treatment (Jemal *et al*, 2008; La Vecchia *et al*, 2009). The obesity epidemic is therefore unlikely to add greatly to the overall burden of cancer.

## Figures and Tables

**Figure 1:**
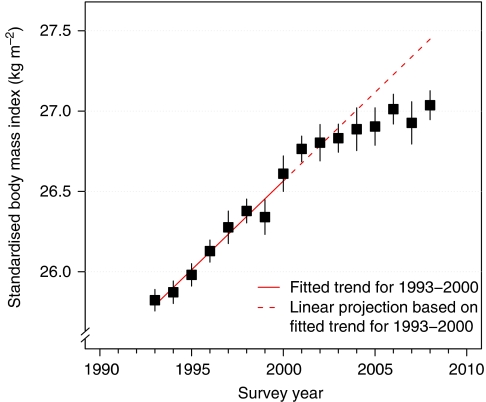
Mean body mass index and 95% CIs standardised by age and sex, for adults in England, 1993–2008. Age- and sex-specific mean values (Joint Health Surveys Unit, 2009) were standardised according to the 2001 Census population (Office for National Statistics, 2001).
